# Identification of pharmacological inhibitors of conventional protein secretion

**DOI:** 10.1038/s41598-018-33378-y

**Published:** 2018-10-08

**Authors:** Liwei Zhao, Peng Liu, Gaelle Boncompain, Friedemann Loos, Sylvie Lachkar, Lucillia Bezu, Guo Chen, Heng Zhou, Franck Perez, Oliver Kepp, Guido Kroemer

**Affiliations:** 1 Faculty of Medicine, University of Paris Sud, Kremlin-Bicêtre, France; 2 Cell Biology and Metabolomics Platforms, Gustave Roussy Cancer Campus, Villejuif, France; 3 Equipe 11 labellisée Ligue Nationale contre le Cancer, Centre de Recherche des Cordeliers, Paris, France; 4 Institut National de la Santé et de la Recherche Médicale, UMR1138, Equipe labellisée Ligue Nationale Contre le Cancer, Paris, France; 5 Université Paris Descartes, Sorbonne Paris Cité, Paris, France; 6 Université Pierre et Marie Curie, Paris, France; 7 Institut Curie, PSL Research University, CNRS UMR144 Paris, France; 8 Pôle de Biologie, Hôpital Européen Georges Pompidou, AP-HP, Paris, France; 9 Department of Women’s and Children’s Health, Karolinska University Hospital, Stockholm, Sweden

## Abstract

The retention using selective hooks (RUSH) system allows to withhold a fluorescent biosensor such as green fluorescent protein (GFP) fused to a streptavidin-binding peptide (SBP) by an excess of streptavidin molecules that are addressed to different subcellular localizations. Addition of biotin competitively disrupts this interaction, liberating the biosensor from its hook. We constructed a human cell line co-expressing soluble secretory-SBP-GFP (ss-SBP-GFP) and streptavidin within the endoplasmic reticulum (ER) lumen and then used this system to screen a compound library for inhibitors of the biotin-induced release of ss-SBP-GFP via the conventional Golgi-dependent protein secretion pathway into the culture supernatant. We identified and validated a series of molecularly unrelated drugs including antianginal, antidepressant, anthelmintic, antipsychotic, antiprotozoal and immunosuppressive agents that inhibit protein secretion. These compounds vary in their capacity to suppress protein synthesis and to compromise ER morphology and Golgi integrity, as well as in the degree of reversibility of such effects. In sum, we demonstrate the feasibility and utility of a novel RUSH-based phenotypic screening assay.

## Introduction

Fluorescence based biosensors introduced into the cells may be observed by (video-) microscopy to detect their abundance as well as their subcellular distribution to obtain morphological information that can be subjected to high-content image analysis^1,2^. Cell-based phenotypic screens allow for the identification of drug effects on living systems without prior knowledge on their molecular targets. Thus, effects on cellular viability, proliferation or organelle homeostasis can be measured in high-throughput screens to identify drugs that act at a high level of potency, followed by target deconvolution^3–5^. Such high-throughput and high-content screens can be designed to obtain sophisticated and detailed functional information on drug effects using human biosensor cell lines. For example, they can be used to characterize drugs with respect to their capacity to modify the cell cycle^6^, to induce cell death by defined pathways such as apoptosis or necroptosis^7,8^, or to stimulate stress responses affecting virtually any organelle^9,10^.

The retention using selective hooks (RUSH) system is based on the reversible interaction between two proteins, the hook and the biosensor^11^. The hook consists in a streptavidin molecule that can be addressed to virtually any subcellular compartment by means of suitable targeting moieties, causing the protein for instance to be withhold in the endoplasmic reticulum (ER) lumen (by means of the KDEL motif) or to be located in other organelles including the Golgi apparatus (GA), mitochondria or the nucleus^11–13^. The biosensor is usually composed of a green fluorescent protein (GFP) - streptavidin binding peptide (SBP) module fused to a secretory cargo such as E-Cadherin, TNF or EGFR. In normal circumstances, this SBP-GFP chimeric protein interacts with streptavidin, meaning that the biosensor is retained by the hook. However, upon addition of biotin, which binds to streptavidin with high affinity (K_d_ = 10^−14^ M), the SBP-GFP tagged protein is released from the hook. Thus, the addition of biotin triggers the close-to-immediate, synchronized release of the SBP-GFP tagged biosensor from streptavidin, allowing the fluorescent cargo to move to another subcellular localization^11^. We have used this system in the past to study the unconventional secretion of the chromatin-binding non-histone protein HMGB1 (high mobility group B1), namely by generating an SBP-GFP-HMGB1 fusion protein that was retained by streptavidin in the nucleus (because streptavidin was fused with several nuclear localization sequences and hence imported in this organelle). Upon addition of biotin plus appropriate HMGB1 secretagogues, the SBP-GFP-HMGB1 biosensor was released first into the cytosol and then outside of the cell, demonstrating the feasibility of the system^13^.

Here, we used the RUSH system to study conventional protein secretion, which is the most frequently used trafficking route that secretory proteins undertake. In this pathway, proteins are transported from the ER to the Golgi apparatus (GA), and subsequently travel to and through the plasma membrane via secretory vesicles^14^. We and others have shown that the RUSH assay can be used to analyze the transport of various conventional secretory cargo^11,15–18^. In this study, we used the simplest reporter that we developed, namely an SBP-GFP fused to a signal peptide in combination with a streptavidin hook addressed to the ER lumen. The biosensor is withheld in the ER until biotin addition: the SBP-GFP biosensor then undertakes the voyage from the ER to the GA and the extracellular space in a highly synchronized, easily quantifiable fashion^14^. We reported in^14^ that the RUSH assay can be adapted to high content screening and we show here that it can efficiently be used to identify and characterize novel inhibitors of conventional protein secretion.

## Results and Discussion

### Screening for pharmacological protein secretion inhibitors by means of the RUSH assay

**Figure 1:**
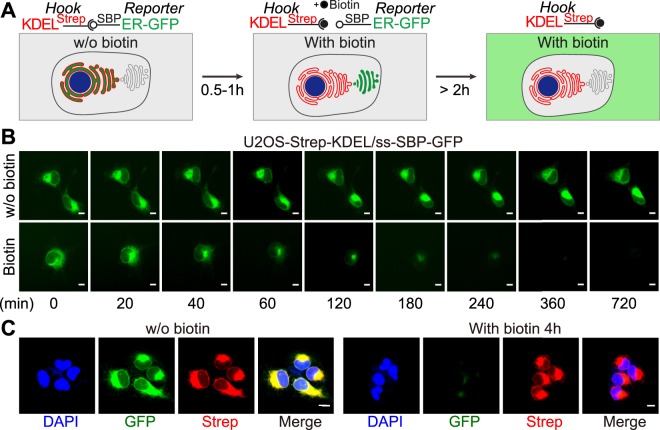
Establishment of the screening system. (**A**) Principle of the RUSH-secretion assay. In the absence of biotin, ss-SBP-GFP reporter is retained in the ER by the streptavidin-KDEL hook. Upon biotin addition, ss-SBP-GFP reporter leaves the ER and accumulates in the Golgi before being released from the cell. (**B**) Time lapse microscopy in the absence or presence of biotin depicts the phenotypic change of cellular florescence. (**C**) Counterstaining and immunostaining of streptavidin in fixed cells preincubated or not with biotin for 4 h. Scale bar equals 10 μm.

We modified a previously described RUSH system to combine (i) an ER-sessile hook (streptavidin-KDEL) expressed by a vector bearing a neomycin resistance cassette with (ii) a GFP reporter that is N-terminally tagged with an SBP domain expressed by a vector conferring hygromycin resistance. The streptavidin-KDEL hook should retain the ss-SBP-GFP reporter in the ER unless excess biotin competitively disrupts this interaction (Fig. 1A). Stable coexpression of both components of the system in human U2OS osteosarcoma cells led to a phenotype of GFP expression in the cytoplasm that was compatible with an ER location. Upon addition of excess biotin to the cells, the GFP fluorescence gradually distributed toward a perinuclear cap, presumably the GA, followed by a strong reduction of the fluorescent signal that was completed around 4 h after the addition of biotin (Fig. 1B). Immunofluorescence detection of streptavidin confirmed that the hook remained in its broadly cytoplasmic distribution upon addition of biotin, confirming that biotin outcompetes the interaction between the ER-sessile hook and the ss-SBP-GFP reporter, allowing for release of the reporter from the cell through the conventional pathway of protein secretion (Fig. 1C). Moreover, counterstaining of the cells to visualize the GA (by immunofluorescence detection of GALT1 or GBF1) confirmed the time-dependent, transient redistribution of ss-SBP-GFP towards the GA after biotin addition (Figs S1 and S2). This redistribution, which normally peaked 20–30 min after addition of biotin, was fully inhibited by the GA-disrupting agent brefeldin A (BFA), which is known to inhibit the anterograde ER-GA protein transport^19^. BFA also suppressed the loss of the ss-SBP-GFP signal from cells after biotin treatment, demonstrating that ss-SBP-GFP release relies on GA-dependent protein secretion (Figs S1 and S2).

**Figure 2:**
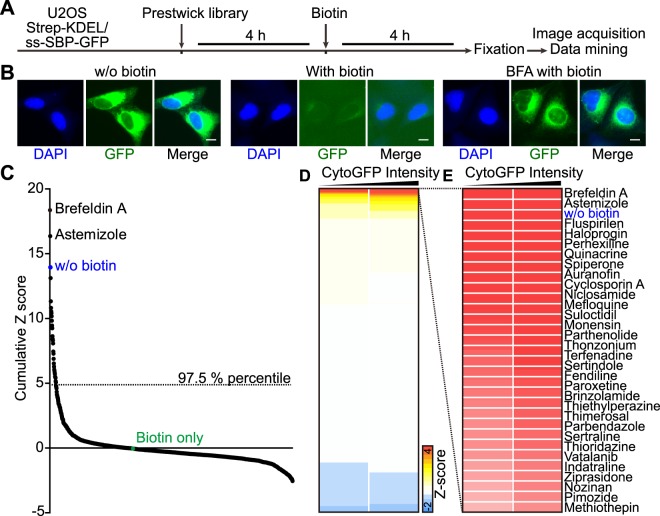
High throughput screening of the Prestwick chemical library. Human osteosarcoma U2OS cells coexpressing streptavidin-KDEL and ss-SBP-GFP were seeded in 384-well plates and treated by the compounds of the Prestwick library at 10 and 20 μM for 4 h. Without removing the drugs, biotin was added at 40 μM and incubated for another 4 h before cells were fixed for image acquisition (**A**). Brefeldin A (BFA) was used as positive control for conventional secretion inhibition. Representative images of cells without GFP release in the absence of biotin, with GFP release in the presence of biotin, as well as with inhibited GFP release by pretreatment of BFA and in the presence of biotin, are depicted in (**B**); scale bar equals 10 μm. Average cytoplasmic GFP intensity was quantified and data was normalized by Z-scoring. Data from two independent experiments were summed up to obtain a cumulative score for each agent (**C**) or were hierarchically clustered and depicted as a heat map in which each block represents the average value obtained for each treatment (mean, n = 4) (**D**). Compounds belonging to the top 2.5% percentile of the resulting ranking are shown.

**Figure 3:**
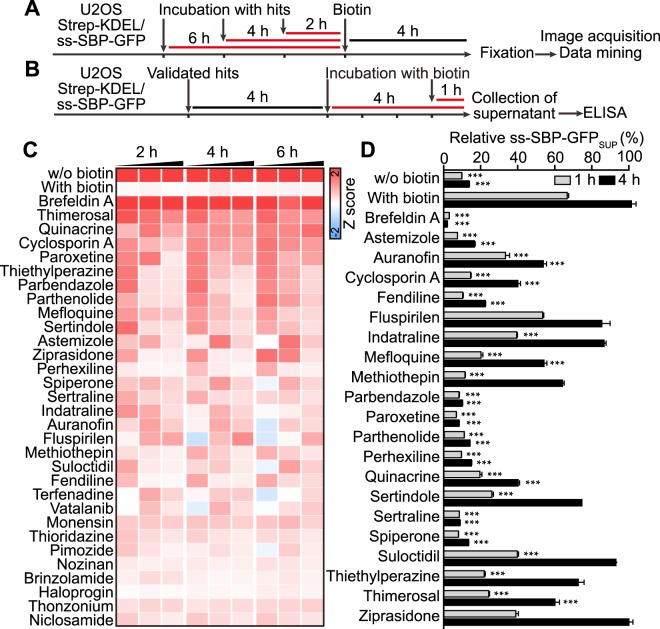
Validation of positive hits identified from the screening. U2OS cells coexpressing streptavidin-KDEL and ss-SBP-GFP were treated with selected agents at 10, 20 and 40 μM for 2, 4, or 6 h before biotin treatment. Four hours after exposure to biotin, cells were fixed for following image acquisition and analysis (**A**). Images were segmented and average cytoplasmic GFP intensity was quantified. The data was normalized by Z-scoring and subjected to hierarchical clustering (**C**). Verified agents were further subjected to an ELISA assay to measure ss-SBP-GFP concentration in supernatants. U2OS coexpressing streptavidin-KDEL and ss-SBP-GFP cells were treated with the selected agents validated as described above at the indicated concentrations for 4 h before addition of biotin, and cell culture supernatants were collected after 1 or 4 h of incubation for ELISA (**B**). The quantity of ss-SBP-GFP in supernatants was normalized to cellular lysates. Cells incubated in the absence of biotin were used as negative controls. Data is reported as mean ± SEM (n = 3) (**D**). Statistical analysis was performed by means of *t* test, ***p < 0.001 as compared to cells treated with biotin alone.

We then used this system to screen the Prestwick library (1200 compounds, all tested at 10 or 20 µM) for the presence of pharmacological agents that inhibit the biotin-induced loss of the GFP signal from the cells (Fig. 2A) while using brefeldin A (BFA) as a positive control of inhibition (Fig. 2B). The screen led to the identification of 30 compounds (2.5% of the library) that inhibited protein secretion by >50% at 10 µM (Fig. 2C–E). We then tested the same compounds again at variable concentrations (10, 20, 40 µM) and pre-incubation periods (2, 4, 6 h before biotin addition) (Fig. 3A,C). Moreover, we developed an enzyme-linked immunosorbent assay (ELISA) that then was used to measure the extracellular accumulation of ss-SBP-GFP induced by addition of biotin for 1 or 4 h, 6 h after preincubation with each of the agents (Fig. 3B,D). Hit compounds were further validated for their effects on the canonical secretion of a GFP reporter in biosensor cells that were released from temperature block at 20 °C by switching them to the normal cell culture temperature of 37 °C (Fig. S3). A further validation step consisted in evaluating their effect on the secretion of interleukin-6 (IL-6) and tumor necrosis factor-α (TNFα) from dendritic cells upon stimulation with lipopolysaccharide (LPS) (Fig. S4). This procedure led to the validation of the protein secretion inhibitory activity of most of the hits from the screening.

### Effects of secretion inhibitors on protein synthesis and ER/GA morphology

We decided to continue the characterization of a few compounds that were selected based on their potency to inhibit ELISA-detectable protein secretion (Fig. 3D), namely, astemizole and parbendazole (two microtubular inhibitors used as anthelmintics), cyclosporine A (an immunosuppressive calcineurin inhibitor), fendiline (a non-selective calcium channel inhibitor acting as a coronary vasodilator), paroxetine and sertraline (two selective serotonin reuptake inhibitors used as antidepressants), parthenolide (a herbal sesquiterpene lactone with multiple effects on signal transduction pathways), perhexiline (an inhibitor of mitochondrial carnitine palmitoyltransferase-1 used as a prophylactic antiaginal agent), quinacrine (an antiprotozoal drug used for the treatment of giardiasis), sertraline (an antipsychotic used for the treatment of schizophrenia) and thimerosal (an antifungal and antiseptic). Thus, a variety of mostly unrelated agents can inhibit protein secretion in U2OS cells (Figs 2–3), as well as in HeLa cells (not shown).

**Figure 4:**
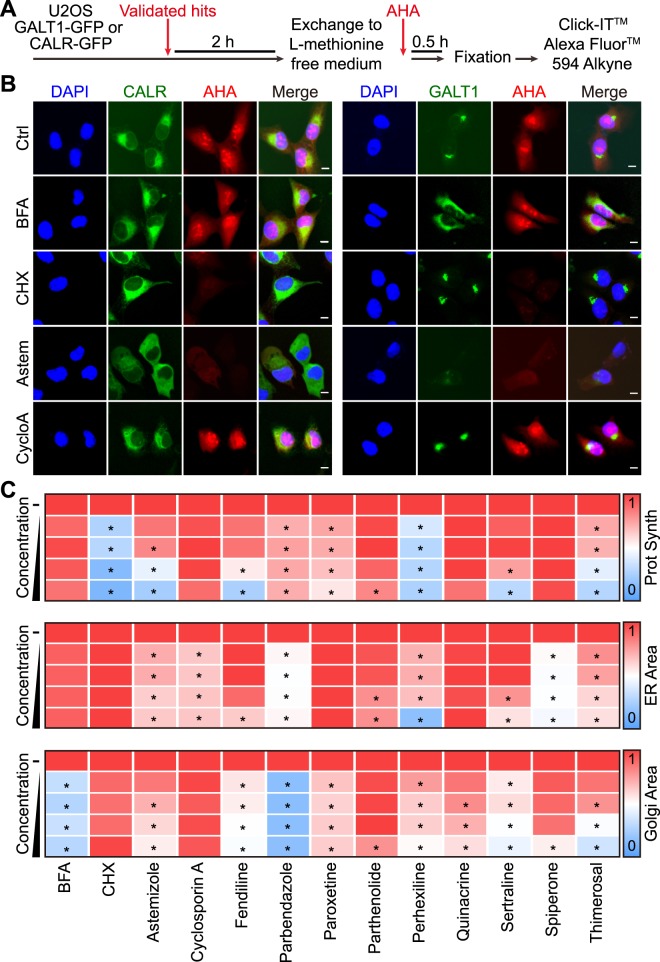
Effects of confirmed secretion inhibitors on the ER and Golgi morphology and global protein synthesis. U2OS cells stably expressing CALR- GFP or GALT1-GFP were pre-treated with the selected secretion inhibitors (5, 10, 20, 40 μM) in L-methionine-free media then incubated with Click-iT^®^ AHA for 30 minutes before subjected to a click reaction with Alexa Fluor^®^ 594 alkyne (**A**). Images were acquired for quantification of nascent protein synthesis (cytoplasmic Alexa Fluor^®^ 594 intensity), ER area (CALR-GFP^high^ area), and Golgi area (GALT1- GFP ^bright^ area). BFA was used as positive control for Golgi disruption, and cycloheximide (CHX) was used as positive control for protein synthesis inhibition. Representative images of untreated controls, positive controls, as well as identified secretion inhibitors with typical ER disruption and/or protein synthesis inhibition (astemizole and cyclosporine **A**) are reported in (**B**), scale bar equals 10 μm. Quantitative data was normalized to untreated control and is summarized as heat map. Each block represents the mean value of 4 repeated measurements (**C**). Statistical analysis was performed by means of multiple *t* test, *p < 0.001 as compared to untreated controls.

**Figure 5:**
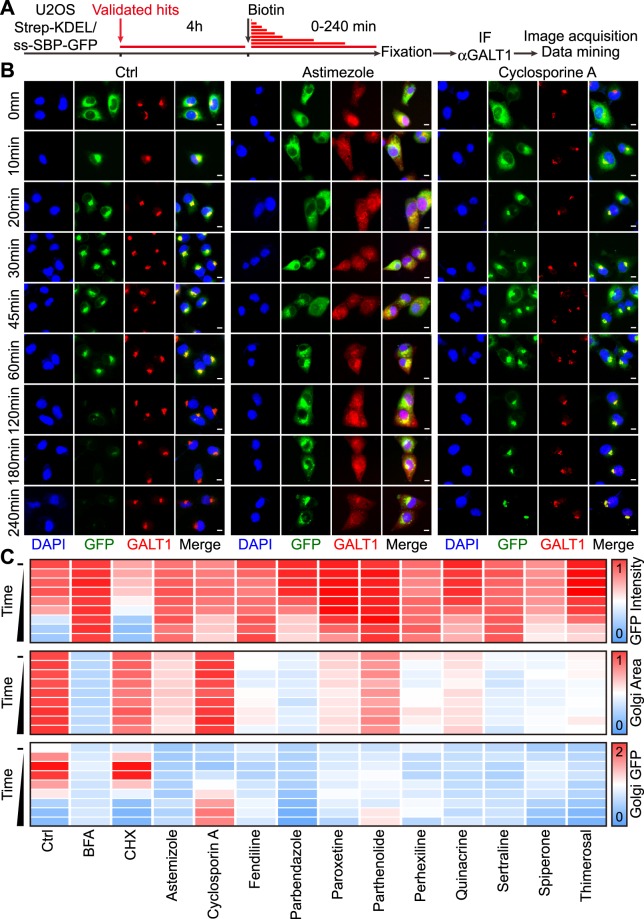
Effects of selected secretion inhibitors on conventional protein transport. U2OS cells coexpressing streptavidin-KDEL and ss-SBP-GFP were treated with selected agents at selected concentrations (20 μM for cyclosporin A, fendiline, parbendazole, paroxetine, parthenolide, quinacrine, sertraline, spiperone, thimerosal; 10 μM for astemizole and perhexiline) for 4 h, followed by biotin addition. Cells were fixed after different incubation periods, followed by immunofluorescence staining of GALT1 (**A**). Cytoplasmic GFP intensity was quantified as a means of protein secretion, GALT1^bright^ area was quantified to indicate Golgi area and Golgi GFP intensity was used to measure the colocalization of GFP-tagged secretory cargo with the Golgi apparatus. Representative images of controls and astemizole or cyclosporine A treated cells are reported in (**B**); scale bar equals 10 μm. Quantitative data was normalized to untreated controls and summarized as heat map. Each block represents the mean value of 4 repeated measurements (**C**).

As a first approximation to the molecular characterization of these agents, we tested them for their capacity to affect the morphology of the ER and GA by adding each of the compounds to U2OS cells stably expressing CALR-GFP or GALT1-GFP fusion proteins in the ER or GA, respectively. In addition, we evaluated the capacity of these agents to inhibit protein synthesis using an analogue of L-methionine, L-azidohomoalanine, which can be detected by click chemistry once it has been incorporated into neosynthesized cellular proteins^20^ (Fig. 4A,B). As positive controls, BFA disrupted the GA without affecting translation, and cycloheximide (CHX) inhibited translation without affecting GA morphology. The protein secretion inhibitors had variable effects on these parameters. For example, perhexiline strongly inhibited translation, while fendiline, parbendazole and sertraline had strong GA-disrupting effects. Moreover, several agents including astemizole and, more so, parbendazole and spiperone led to a change in the distribution of the ER network that became more diffuse (Fig. 4B,C). We also investigated the time-dependent, biotin-induced relocation of the ss-SBP-GFP reporter in the RUSH assay, both in control conditions and in the presence of each of the drugs of the panel. All the chosen agents interfered with the loss of the GFP intensity, with BFA as a positive and CHX as a negative control. This latter result indicates that inhibition of protein translation is not sufficient to suppress protein secretion. Note that the effect seen on ss-SBP-GFP secretion cannot be directly attributed to a reduction of cargo neosynthesis as it is accumulated in the ER before the addition of the drugs. The majority of the agents selected from the screen caused the disintegration or dissipation of the GA, as exemplified for astemizole, fendiline, parbendazole, perhexiline, sertraline, spiperone and thimerosal. However, cyclosporine A and parthenolide barely affected the morphology of the GA. In contrast, both agents, in particular cyclosporine A, apparently slowed down the transit of the ss-SBP-GFP reporter towards the GA. This move, which can be quantified as the colocalization between ss-SBP-GFP and the GALT1-dependent red fluorescent signal, is maximal in controls around 20–30 min after biotin addition yet occurs much later in the presence of cyclosporine A, where it peaks at 180–240 min (Fig. 5A–C).

Altogether, we conclude from these results that the agents inhibiting protein secretion have rather distinct modes of action, commensurate with the fact that they fall into rather distinct drug classes.

### Reversibility of secretion inhibitor effects

**Figure 6:**
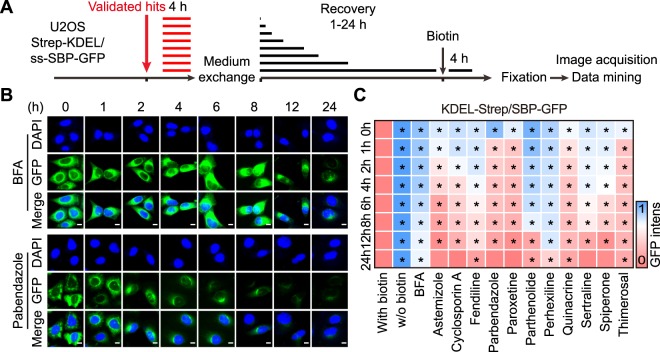
Study on the reversibility of selected agents on protein secretion inhibition. U2OS cells coexpressing streptavidin-KDEL and ss-SBP-GFP were treated with selected agents at selected concentrations (20 μM for cyclosporin A, fendiline, parbendazole, paroxetine, parthenolide, quinacrine, sertraline, spiperone, thimerosal; 10 μM for astemizole and perhexiline) for 4 h. Then the drugs were washed out and replaced with fresh medium for cells to recover from the treatment. After different recovery periods, cells were incubated with biotin for another 4 h before fixation and image acquisition (**A**). Representative images of BFA (which is relatively irreversible) pretreated cells as well as parbendazole (which is highly reversible) treated cells after different recovery periods are reported in (**B**), scale bar equals 10 μm. Quantitative data was normalized to untreated controls and summarized as heat map, each block represents the mean value of 4 repeated measurements (**C**). Statistical analysis was performed by means of multiple *t* test, *p < 0.001 as compared to untreated controls.

**Figure 7:**
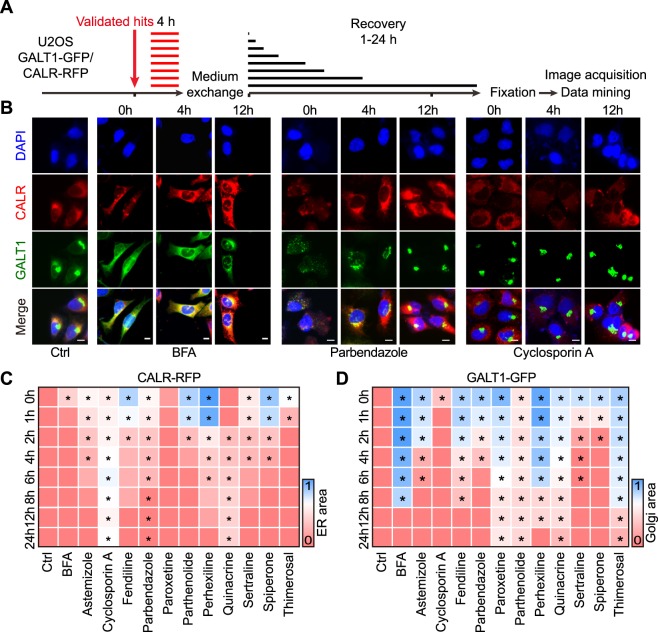
Study on the reversibility of selected agents on ER and Golgi disruption. U2OS cells stably coexpressing GALT1-GFP and CALR-RFP were pre-treated by the selected secretion inhibitors at selected concentrations (20 μM for cyclosporin A, fendiline, parbendazole, paroxetine, parthenolide, quinacrine, sertraline, spiperone, thimerosal; 10 μM for astemizole and perhexiline) for 4 h. Then the drugs were washed out and replaced with fresh medium for cells to recover from treatments. After different recovery periods, the cells were fixed for image acquisition and analysis (**A**). Representative images of control cells and BFA, parbendazole, and cyclosporine A treated cells after different recovery periods are reported (**B**), scale bar equals 10 μm. Quantitative data was normalized to untreated controls and summarized to a heat map. Each block represents the mean value of 4 repeated measurements (**C**,**D**). Statistical analysis was performed by means of multiple *t* test, *p < 0.001 as compared to untreated controls.

In the next step, we investigated the putative reversibility of the drug effects on protein secretion and organellar morphology. The biosensor cells were cultured for 4 h in the presence of the distinct agents, then washed, allowed to recover for a variable period (1 to 24 h) and finally subjected to the RUSH assay (Fig. 6A). When BFA was washed out, this led to a gradual, though only partial recovery of protein secretion, meaning that at 24 h post BFA removal the cells still exhibited a significant inhibition of ss-SBP-GFP secretion (Fig. 6B). Several among the inhibitors allowed for full recovery of protein secretion at 24 h post washout; this applies to astemizole, cyclosporine A, parbendazole, paroxestine, sertraline and spiperone but not the remaining compounds. Moreover, the speed of recovery appears to be quite heterogeneous with a rapid recovery for parbendazole, paroxetine and thimerosal (detectable after 2 h of washout), but slower kinetics for fendiline and sertraline (detectable after 6 h of washout) (Fig. 6B,C). We performed similar washout experiments to assess the reversibility of ER and GA perturbation measurable in cells co-expressing CALR-RFP and GALT1-GFP (Fig. 7A,B). This approach again revealed a major heterogeneity in the recovery rates after the removal of each of the different inhibitors, as determined for the ER (Fig. 7C) and the GA (Fig. 7D). These results were confirmed in wild type U2OS and in HeLa cells by immunofluorescence detection of endogenous CALR and GALT1, yielding a similar pattern of variable recovery for each of the distinct secretory inhibitors (Fig. S5, S6). Altogether, these results support a major heterogeneity in the mode of action of the however limited panel of compounds subjected to in-depth characterization.

### Concluding remarks

**Figure 8:**
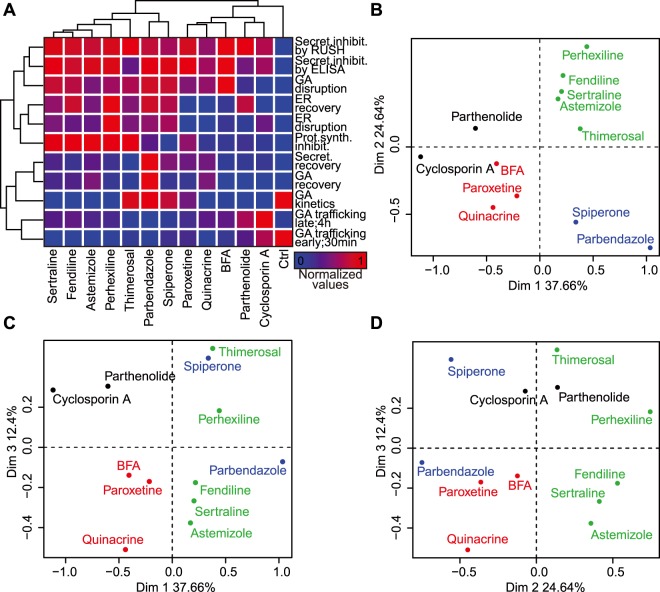
Hierarchical clustering by principal component analysis. (**A**) Data were compiled and standardized between 0 and 1 before being subjected to hierarchical clustering. Results are depicted as heatmap. (**B**–**D**). Dot plots represent the projection coordinates of 3 retained dimensions from principal component analysis. Each of the 4 groups stemming from k-means clustering is shown as a different color.

The aforementioned results, all of which have been obtained by automated image analysis, were subjected to a bioinformatic evaluation designed to classify the agents in different groups. For this, each of the measured parameters (inhibition of ELISA-detectable protein secretion, inhibition of the RUSH assay and its recovery, GA dissipation and recovery, ER disruption and recovery, co-localization of the ss-SBP-GFP reporter with GA measured at 30 min and 4 h or as a kinetics) were reduced to mono-dimensional numeric values to quantify the effects of each of the compounds. These data were subjected to correlation analyses (Fig. S7), non-supervised hierarchical clustering (Fig. 8A), and principal component analysis (Fig. 8B–D, Fig. S8). This latter procedure led to the identification of 4 clusters of agents, namely, a cluster of 5 compounds (astemizole, fendiline, perhexilline, sertraline, thimerosal), one of 2 (spiperone and parbendazole), one of 3 compounds (brefeldin A, paroxeten, quinacrine) and yet another one of 2 agents (cyclosporine A and parthenolide) (Fig. 8B–D). Hence, some of the agents that are closely related in their mode of action (like the antidepressants fendiline and sertraline) fall into the same cluster, while others (like the anthelmintics astemizole and parbendazole) separate into distinct clusters. Nevertheless, STITCH analysis did not reveal common interacting protein clusters (Fig. S9).

In conclusion, we built a high throughput/high content screening system for the discovery and characterization of protein secretion-modulatory agents. This system allows to identify agents that interfere with the movement of proteins from the ER towards the GA and then outside of the cell. Within a compound library, this screening method led to the detection of protein secretion-inhibitory drugs that previously were not suspected to have such effects. Clinically used drugs were found to heterogeneously perturb protein synthesis and ER/GA morphology, with a variable degree of reversibility, indicating that they likely differ in their mode of action. It remains to be seen whether such unsuspected effects may be achieved at clinically relevant concentrations or when they these drugs are overdosed (for instance in the context of suicidal ingestion), perhaps explaining unwarranted and toxic effects of some of these agents. Irrespective of this open question, it appears that the screening system may be amenable to the identification of novel protein secretion inhibitors, as well as – at least theoretically – new secretagogues.

## Materials and Methods

### Cell culture, chemicals and antibodies

Human osteosarcoma U2OS cells and human cervical cancer HeLa cells were cultured in Dulbecco’s Modified Eagle Medium (DMEM) in supplement with fetal bovine serum (FBS, 10% v/v), penicillin (100 U/mL), and streptomycin (100 μg/mL) in a humidified environment containing 5% CO_2_ at 37 °C. All cell culture related products were from Gibco-Invitrogen (Carlsbad, CA, USA) and all plastic materials were obtained from Corning (Corning, NY, USA). The Prestwick chemical library was obtained from Prestwick Chemical (Illkirch, France) and aliquoted for stable storage. Small molecules for experiments other than screening were purchased from Sigma-Aldrich (St Louis, MI, USA). Selection antibiotics including Geneticin (G418) and hygromycin (Hygro) were purchased from Invivogen (Eugene, OR, USA). Primary antibodies to streptavidin (clone S10D), SBP tag (clone SB19-C4) were obtained from Santa Cruz Biotechnology (Dallas, TX, USA); GFP (ab6556), GBF1 (ab86071) were obtained from Abcam (Cambridge, UK); B4GALT1 (PAB20512) was from Abnova (Taipei, China). Alexa Fluor® conjugated secondary antibodies were purchased from Invitrogen (Carlsbad, CA, USA).

### Construction of the RUSH protein secretion system

U2OS cells were first transduced with lentiviral particles packaged with the streptavidin-KDEL plasmid to establish ER hook expressing cell lines. Cells were selected with G418 for 2 weeks and sorted in 96-well plates for cloning. Clones were selected by means of streptavidin immunofluorescence. U2OS streptavidin-KDEL stably expressing cells were transduced with lentiviral particles packaged with ss-SBP-GFP plasmid and subsequently selected with hygromycin and G418 before cloning. For the generation of ss-SBP-GFP pCDH was modified to remove the EF1 promoter and the puromycin resistance cassette driven by EF1 promoter. An IRES and a hygromycin resistance cassette were introduced downstream of SBP-EGFP. Clones were functionally validated in the presence of biotin by visual inspection.

### Immunofluorescence staining of fixed cells

Cells were seeded in black µclear 384-well imaging plates (Greiner bio one, Kremsmünster, Austria) and cultured for 24 h before treatment with selected agents or biotin, followed by washing and fixation with 4% paraformaldehyde (PFA, Sigma) containing 2 µg/mL Hoechst 33342 (Life Technologies, Carlsbad, CA, USA) for 30 min at room temperature (RT). Cells were then washed and permeabilized with 0.5% Triton X-100 (Sigma) for 15 min at RT. Permeabilized cells were blocked with 2% BSA for 1 h before incubation with the primary antibody diluted in PBS containing 1% BSA overnight at 4 °C. Cells were washed several times and following incubated with Alexa Fluor® conjugated secondary antibody dilution containing 1% BSA for 1 h at RT. Finally, cells were washed and plates were sealed with aluminum adhesive cover for image acquisition.

### High-throughput screening for secretory pathway inhibitors

U2OS Strep-KDEL/ss-SBP-GFP cells were seeded in black µclear 384-well imaging plates and cultured for 24 h. On the second day, compounds from the Prestwick library were added to cells at two different concentrations (10, 20 µM), BFA (10 µg/mL) was used as a positive control for the inhibition of protein secretion. Cells were incubated with the compounds for 4 h before the addition of biotin (40 µM) untreated cells were used as controls. Following incubation of the cell for 4 h, medium was discarded and cells were fixed with 4% PFA containing 2 µg/mL Hoechst 33342 overnight at 4 °C. Next the cells were washed, superseded with 50 μL of PBS and subjected to automated image acquisition and subsequent image analysis. For the automated acquisition of fluorescent signal, a robot-assisted IXM XL BioImager (Molecular Devices, Sunnyvale, CA, USA) equipped with a Sola light source (Lumencor, Beaverton, OR, USA), adequate excitation and emission filters (Semrock) a 16-bit monochrome sCMOS PCO.edge 5.5 camera (PCO, Kelheim, Germany) and a 20 X PlanAPO objective (Nikon, Tokyo, Japan) was used to acquire a minimum of 4 view fields in each well. The acquired images were processed with the MetaXpress image analysis software (Molecular Devices). Images were segmented using the built-in custom module editor to identify nucleic, and cytoplasmic regions, allowing for the quantification of cytoplasmic GFP intensity. Data were mined and statistically evaluated using the freely available software R (https://www.r-project.org). The average cytoplasmic GFP intensity of each sample was normalized to control wells incubated with biotin and thereafter used to determine Z-scores.

### Sandwich ELISA for measurement of cell culture supernatant ss-SBP-GFP quantity

U2OS Strep-KDEL/ss-SBP-GFP cells were seeded in 24-well plates and let adapt for 24 h. Following cells were treated with the selected agents or BFA for 4 h, followed by further incubation with biotin for another 1 or 4 h. Cell culture supernatants were collected for ELISA. Cells from an additional well (without biotin) from each plate were harvested in original medium and lysed by ultrasound, followed by centrifugation at >5,000 g for 10 min at 4 °C to remove debris, and a serial dilution of supernatant served as standards. For the ELISA assay, high-binding 96-well plates (Corning) were coated with a mouse anti-SBP antibody diluted PBS overnight at 4 °C. The coated plates were then washed with PBST (PBS containing 0.05% Tween-20) and blocked with PBS containing 2% BSA for 1 h at RT, then the blocking buffer was replaced with 100 µl PBST containing 1% BSA/PBST. Supernatants and standards were added to the plates (10 μL/well) and incubated overnight at 4 °C. Solutions were discarded and wells were repeatedly washed with PBST before incubation with rabbit anti-GFP antibody diluted in 100 μL 1% BSA containing PBST for 2 h at RT. Following solutions were discarded and wells were repeatedly washed with PBST before incubation with a HRP-conjugated goat-anti-rabbit secondary antibody diluted in 100 μL 1% BSA containing PBST) for 1 h at RT. Solutions were discarded and wells were repeatedly washed with PBST before incubation with a 1-Step Ultra TMB-ELISA Substrate Solution (Life Technologies), followed by incubation with 50 μL 2 M sulfuric acid. Absorbance of each well was measured at 450 nm with a SpectraMax multi-mode microplate reader (Molecular Devices).

### Click-iT^®^ AHA Alexa Fluor^®^ 594 Protein Synthesis Assay

The Click chemistry assay to measure protein synthesis was adapted from the *Click-iT*^*®*^ *AHA Alexa Fluor*^*®*^ *488 Protein Synthesis HCS Assay* (C10289, Invitrogen). Generally, cells were seed in 384-well plates (2000 cells/well) and let adapt for 24 h. Agents for cell treatment were prepared in L-methionine free medium contains 5% L-methionine free FBS, 2 mM L-glutamine, 10 mM HEPES, and 200 μM L-cystine (all from Invitrogen) and added to cells. After incubation of 2 h, old medium was replaced with 25 μL fresh L-methionine free medium as described above, containing 50 μM Click-IT™ AHA (L-Azidohomoalanine, C10102, Invitrogen) and incubated for additional 30 min. After incubation, medium containing Click-iT^®^ AHA was removed and cells were washed once with PBS before fixation with 4% PFA containing 2 µg/mL Hoechst 33342 for 20 min at RT. Next, cells were washed twice with 2% BSA in PBS and permeabilized with 0.5% Triton^®^ X-100 in PBS for 20 minutes at RT. After additional washing steps with 2% BSA in PBS, cells were incubated with the Click-iT^®^ reaction cocktail composed of Alexa Fluor™ 594 Alkyne (A10275, Invitrogen, 0.5 µM), CuSO4 (2 µM), Click-iT® AHA buffer additive (Component C, from C10289, Invitrogen, 2 mg/mL), dissolved in TBS. The cells were incubated for 30 min at RT in the dark, followed by additional washing steps with 2% BSA in PBS before image acquisition.

### Bioinformatics analysis

After standardization to controls, the dataset was subjected to a principal component analysis (PCA), from which 3 dimensions were retained, cumulating 75% of the inertia. A k-means clustering was applied to these 3 dimensions, differentiating 4 distinct groups.

### Statistical analysis

Unless otherwise specified, data are reported as mean ± SEM of a minimum of three independent experiments. Statistical significance was analyzed using the Student’s-test. Differences to control cells or between groups as indicated were considered to be significant if *p < 0.05, **p < 0.01 ***p < 0.001.

## Electronic supplementary material

Supplementary Information
