# Supplementary material for: Identification of pharmacological inhibitors of conventional protein secretion
Source: Sci Rep. 2018 Oct 8;8:14966. doi: 10.1038/s41598-018-33378-y (PMC6175952; doi:10.1038/s41598-018-33378-y)
Supplement: Supplementary file 1 — Supplementary Information [file 41598_2018_33378_MOESM1_ESM.pdf]

Supplemental files

Identification of pharmacological inhibitors of conventional protein secretion

Liwei Zhao[1-6&], Peng Liu[1-6&], Gaelle Boncompain[7&], Friedemann Loos[2-6], Sylvie Lachkar[2-6], Lucillia Bezu[2-6], Guo Chen[2-6], Heng Zhou[1-6], Franck Perez[7*], Oliver Kepp[1-6*], and Guido Kroemer[2-6,8,9*]

[1]Faculty of Medicine, University of Paris Sud, Kremlin-Bicêtre, France; [2]Cell Biology and Metabolomics Platforms, Gustave Roussy Cancer Campus, Villejuif, France; [3]Equipe 11 labellisée Ligue Nationale contre le Cancer, Centre de Recherche des Cordeliers; Paris, France; [4]Institut National de la Santé et de la Recherche Médicale, UMR1138, Equipe labellisée Ligue Nationale Contre le Cancer, Paris, France; [5]Université Paris Descartes, Sorbonne Paris Cité, Paris, France; [6]Université Pierre et Marie Curie, [7]Institut Curie, PSL Research University, CNRS UMR144, Paris, France; [8]Pôle de Biologie, Hôpital Européen Georges Pompidou, AP-HP; Paris, France; [9]Department of Women's and Children's Health, Karolinska University Hospital, Stockholm, Sweden.

[&] LZ, PL and GB share first co-authorship.

* FP,OK and GK are senior co-authors of this paper.

Correspondence to: **Dr. Guido Kroemer** kroemer@orange.fr

Dr. Oliver Kepp captain.olsen@gmail.com

Key Words: endoplasmic reticulum, Golgi apparatus, screening system

Authors contribution: L.Z, P.L, F.L, S.L, H.Z, L.B, G.C performed experiments; G.B and F.P provided the RUSH assay; O.K and G.K conceived the study; L.Z, P.L, O.K and G.K wrote the paper.

Conflicts of interest: The authors declare no conflict of interest.

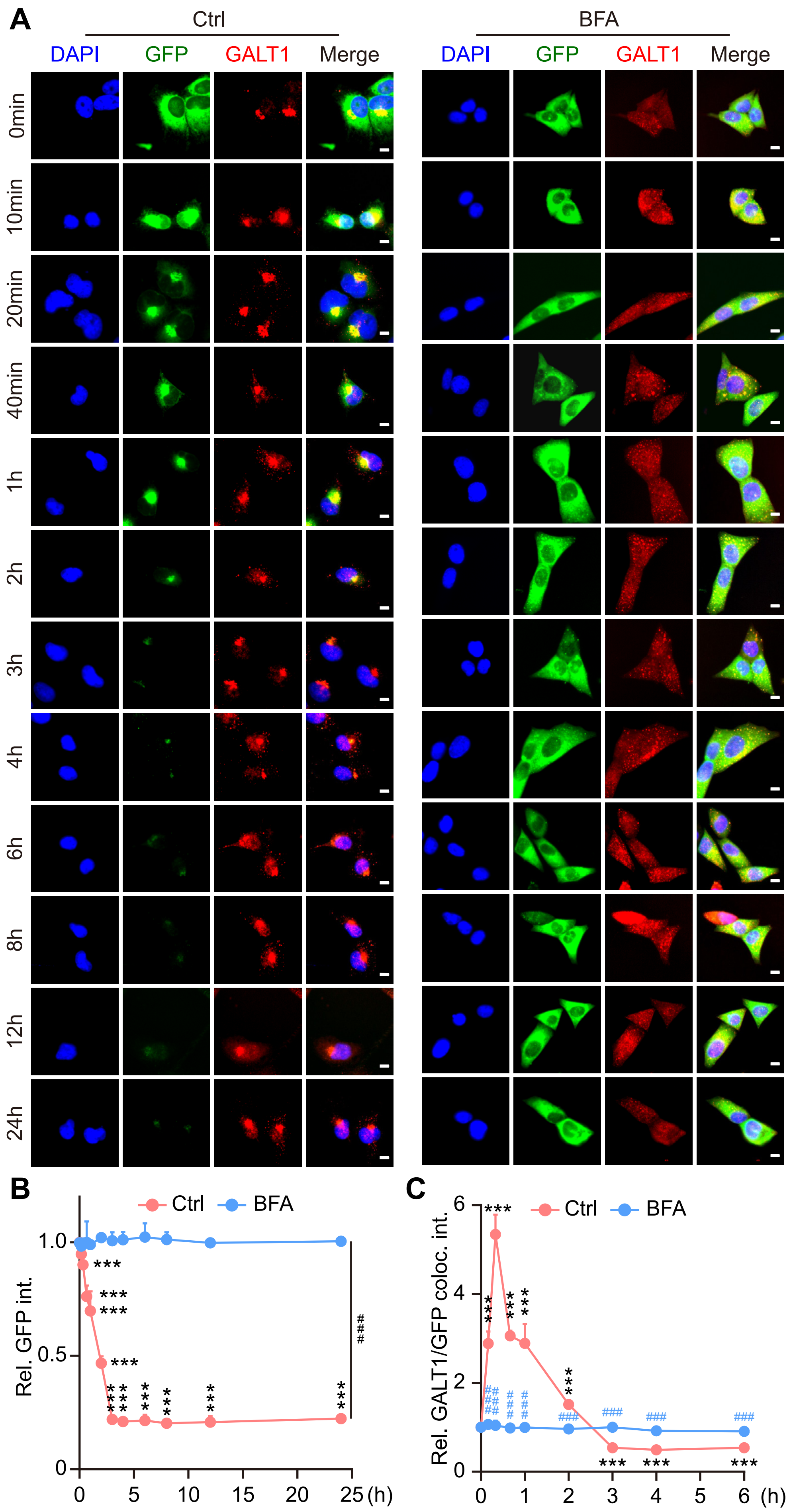

Figure S1. Characterization of the RUSH protein secretion assay. U2OS cells coexpressing streptavidin-KDEL and ss-SBP-GFP were pretreated or not with the classical protein secretion inhibitor brefeldin A (BFA) followed by incubation with biotin for different periods before fixation for immunofluorescence with a GALT1 antibody for Golgi apparatus labeling. Representative images (**A**) as well as quantified cytoplasmic GFP intensity (**B**) and Golgi localized GFP intensity (**C**) are reported. Statistical analysis was performed by means of multiple t test, ***p<0.001 as compared to values at 0 h; ###p<0.001 as comparing between Ctrl and BFA at the same time points.

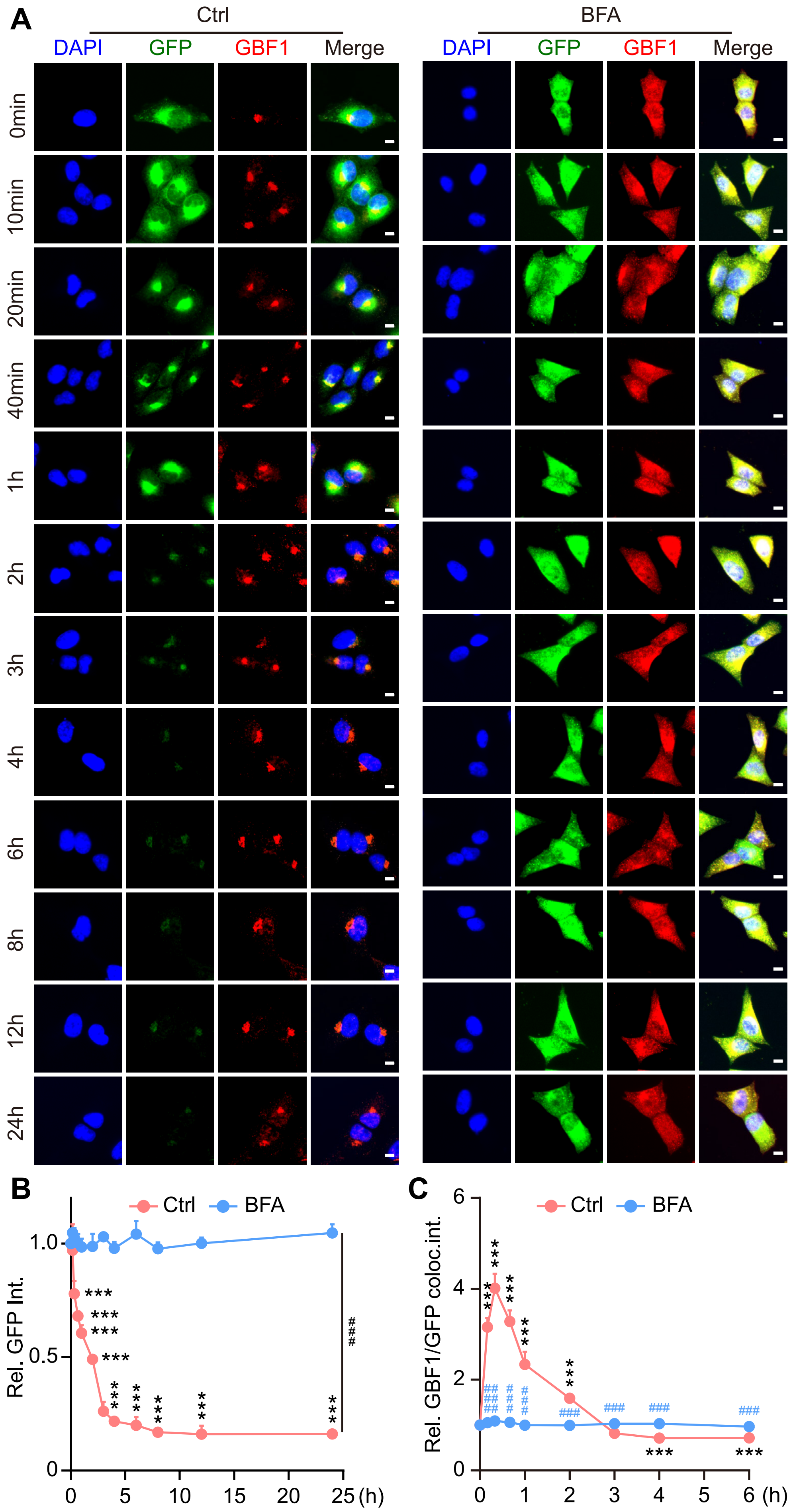

Figure S2. Characterization of the RUSH protein secretion assay. U2OS cells coexpressing streptavidin-KDEL and ss-SBP-GFP were pretreated or not with classical protein secretion inhibitor brefeldin A (BFA) before biotin was added for different periods and the cells were fixed for immunofluorescence with a GBF1 antibody for Golgi apparatus labeling. Representative images (**A**) as well as quantified cytoplasmic GFP intensity (**B**) and Golgi localized GFP intensity (**C**) are reported. Statistical analysis was performed by means of multiple t test, ***p<0.001 as compared to values at 0 h; ###p<0.001 as comparing between Ctrl and BFA at the same time points.

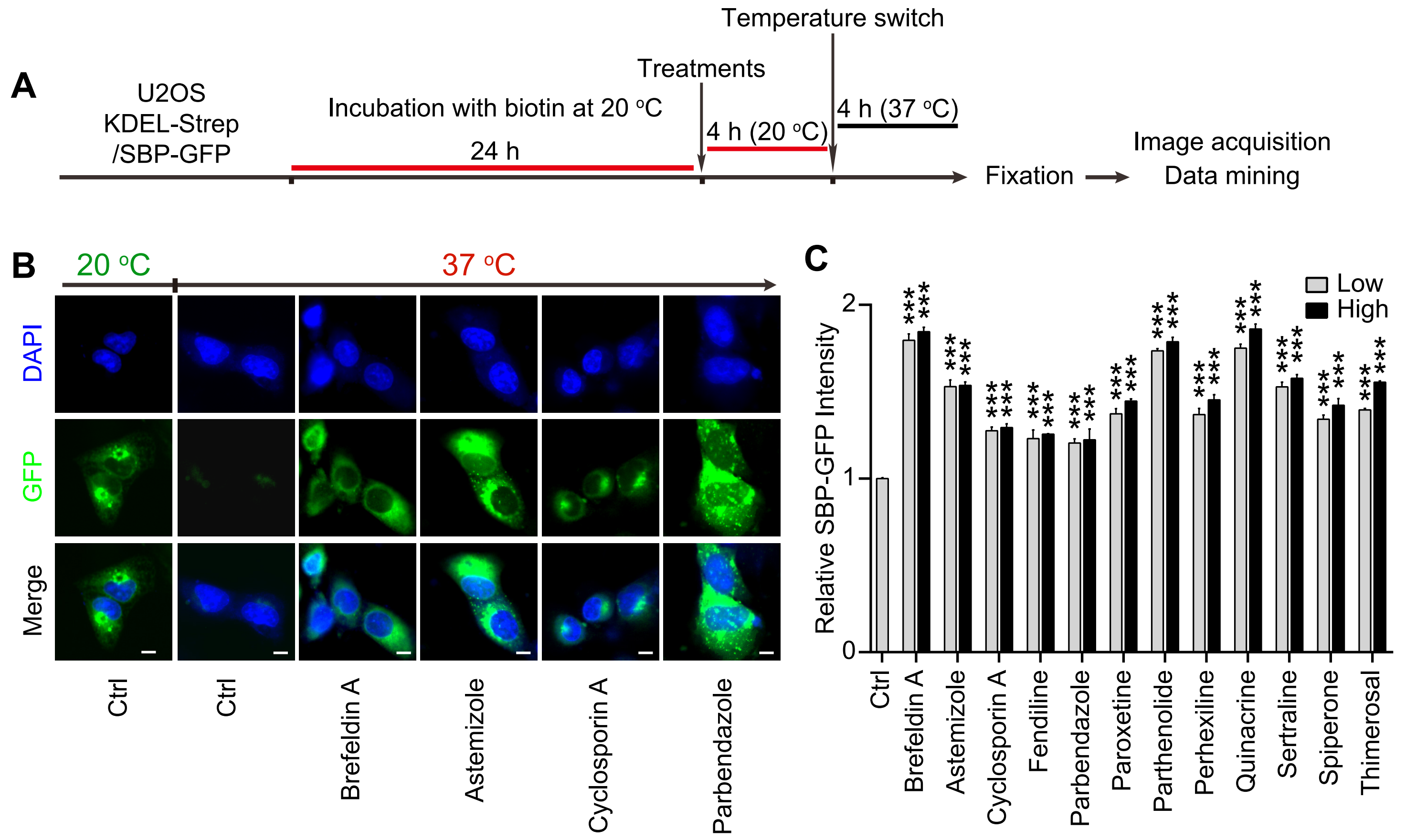

Figure S3. Validation of secretion inhibitors by temperature shift assay. U2OS cells co-expressing KDEL-Streptavidin and SBP-GFP were seeded in 384-well plates and kept in the continuous presence of biotin to induce secretion of the reporter. Cells were preincubated at 20°C to block canonical protein secretion before addition of selected inhibitors (at two different concentrations) for 4 h. Then the cells were transferred to, and maintained at, 37°C for additional 4 h to (re-)activate protein secretion before fixation and image acquisition. Untreated controls were imaged before and after temperature shift to depict the efficacy of the applied temperature block (**A**). Representative images are shown in (**B**). Images were segmented and average cytoplasmic GFP intensity was quantified. Data was normalized to the average value of untreated wells and is depicted as mean ± SEM (n=4) (**C**). Statistical analysis was performed by means of the Student's t test, ***p<0.001 as compared to untreated control.

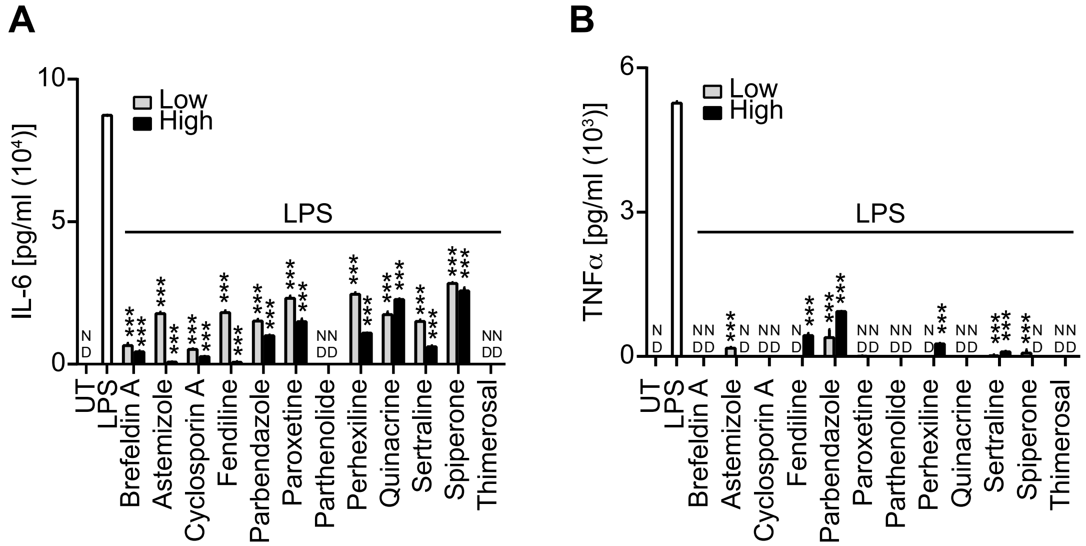

Figure S4. Effects of selected inhibitors on cytokine secretion from dendritic cells (DC). Induced-immortalized DCs (iniDCs) were de-induced as described in the Materials and Methods to obtain de-iniDCs that phenocopy the secretory functions of primary DCs. After 3 days de-iniDCs were treated with selected inhibitors (at two different concentrations) for 4 h before stimulation with LPS for additional 24 h. Secretion of IL-6, and TNFα into the supernatant was measured by ELISA (**A,B**). Data is shown as mean ±SEM (n=3). Statistical analysis was performed by means of the t test, ***p<0.001 as compared to wells only stimulated with LPS, ND not detected.

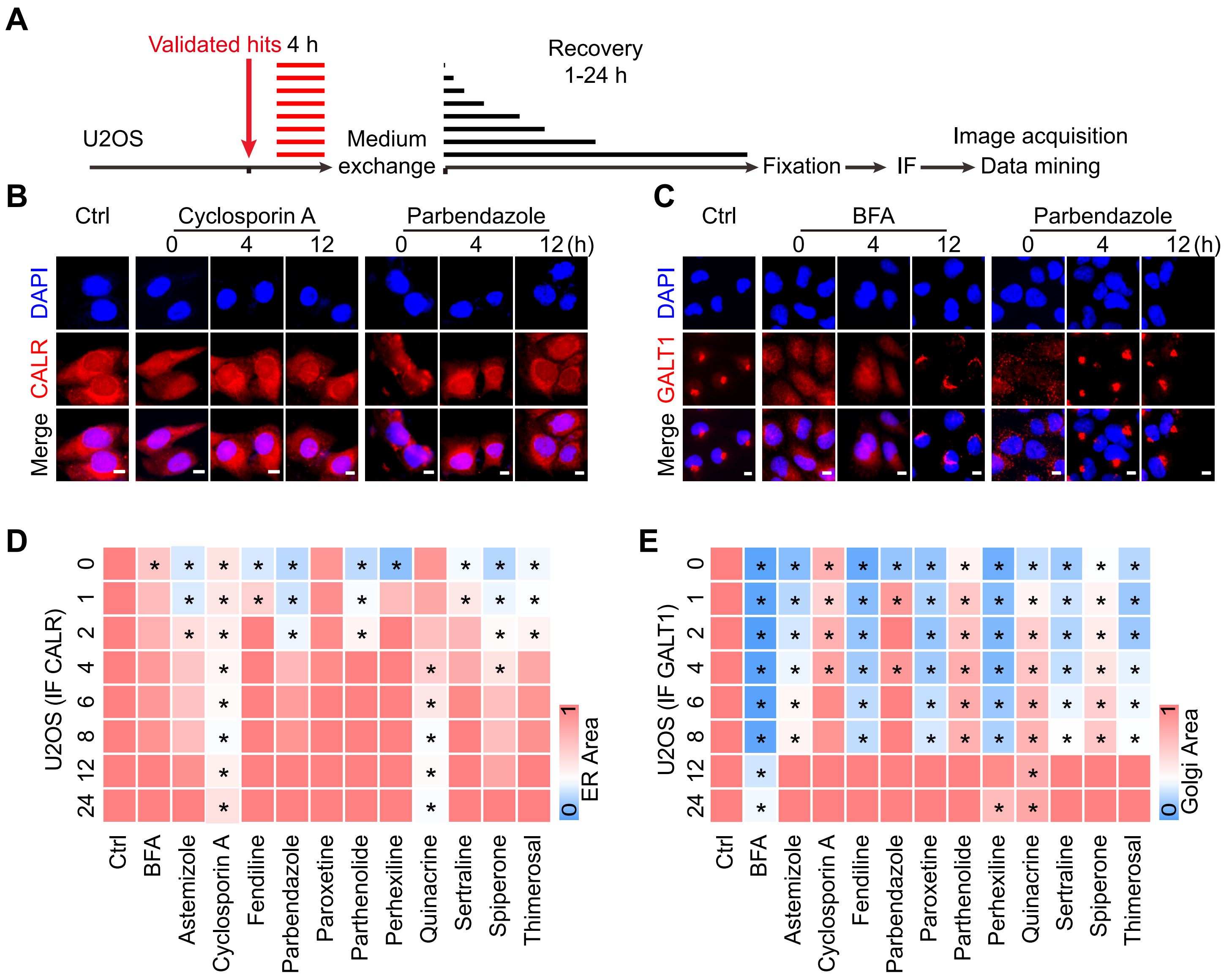

Figure S5. Reversibility of ER and Golgi disruption induced by selected agents in U2OS. Human osteosarcoma U2OS cells were pre-treated with the selected secretion inhibitors at selected concentrations (20 µM for cyclosporin A, fendiline, parbendazole, paroxetine, parthenolide, quinacrine, sertraline, spiperone, thimerosal; 10 µM for astemizole and perhexiline) for 4 h. Then drugs were washed out and replaced with fresh medium for cells to recover from the treatment. After different recovery period, cells were fixed and subjected to immunofluorescence of CALR and GALT1 (**A**). Representative images of control cells, and cyclosporine or parbendazole treated cells after different recovery periods are depicted to exemplify the recovery of ER fragmentation (**B**). BFA or parbendazole treated cells are shown to exemplify typical recovery from Golgi disruption (**C**), scale bar equals 10 µm. Quantitative data was normalized to untreated controls and summarized as heat map, each block represents the mean value of 4 repeated measurements (**D, E**). Statistical analysis was performed by means of multiple t test, *p<0.001 as compared to untreated controls.

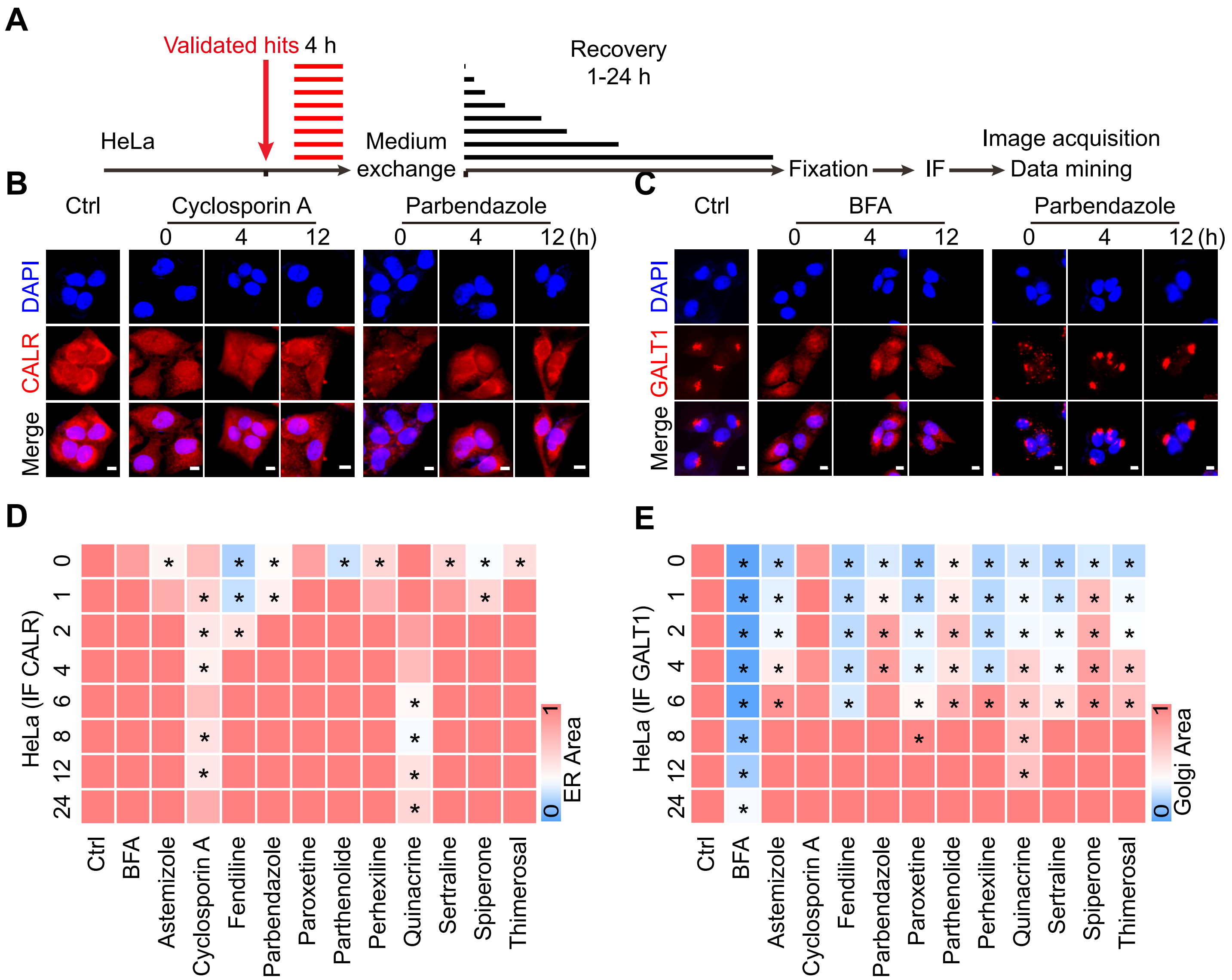

Figure S6. Reversibility of ER and Golgi disruption induced by selected agents in HeLa. Human cervix carcinoma HeLa cells were pre-treated with the selected secretion inhibitors at selected concentrations (20 µM for cyclosporin A, fendiline, parbendazole, paroxetine, parthenolide, quinacrine, sertraline, spiperone, thimerosal; 10 µM for astemizole and perhexiline) for 4 h. Then drugs were washed out and replaced with fresh medium for the cells to recover from treatments. Following different recovery periods, cells were fixed and subjected to immunofluorescence of CALR and GALT1 for image acquisition and analysis (**A**). Representative images of control cells, and cyclosporine or parbendazole treated cells after different recovery periods are depicted to exemplify the recovery of ER fragmentation (**B**). BFA or parbendazole treated cells are shown to exemplify typical recovery from Golgi disruption (**C**), scale bar equals 10 µm. Quantitative data was normalized to untreated controls and summarized as heat map, each block represents the mean value of 4 repeated measurements (**D, E**). Statistical analysis was performed by means of multiple t test, *p<0.001 as compared to untreated controls.

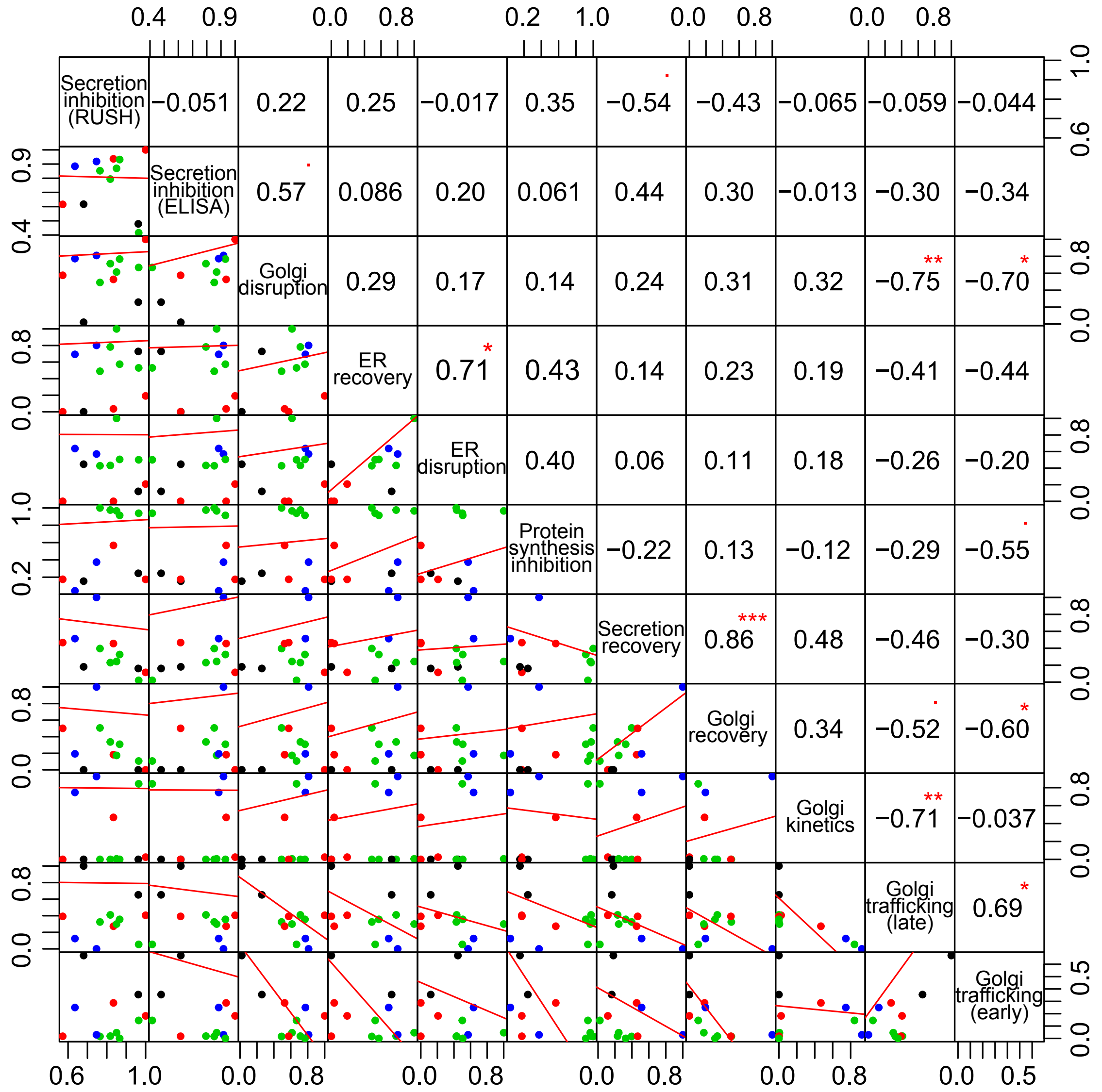

Figure S7 Systematic correlation analysis between descriptive variables. Correlations between standardized variables were calculated pairwise and are reported as a correlation matrix. The upper panel indicates the calculated Spearman correlation coefficients and significance (*p<0.05, **p<0.01, ***p<0.001), the lower panel shows bi-parametric dot plot with regression line. Colors represent groups as described in Fig. 8.

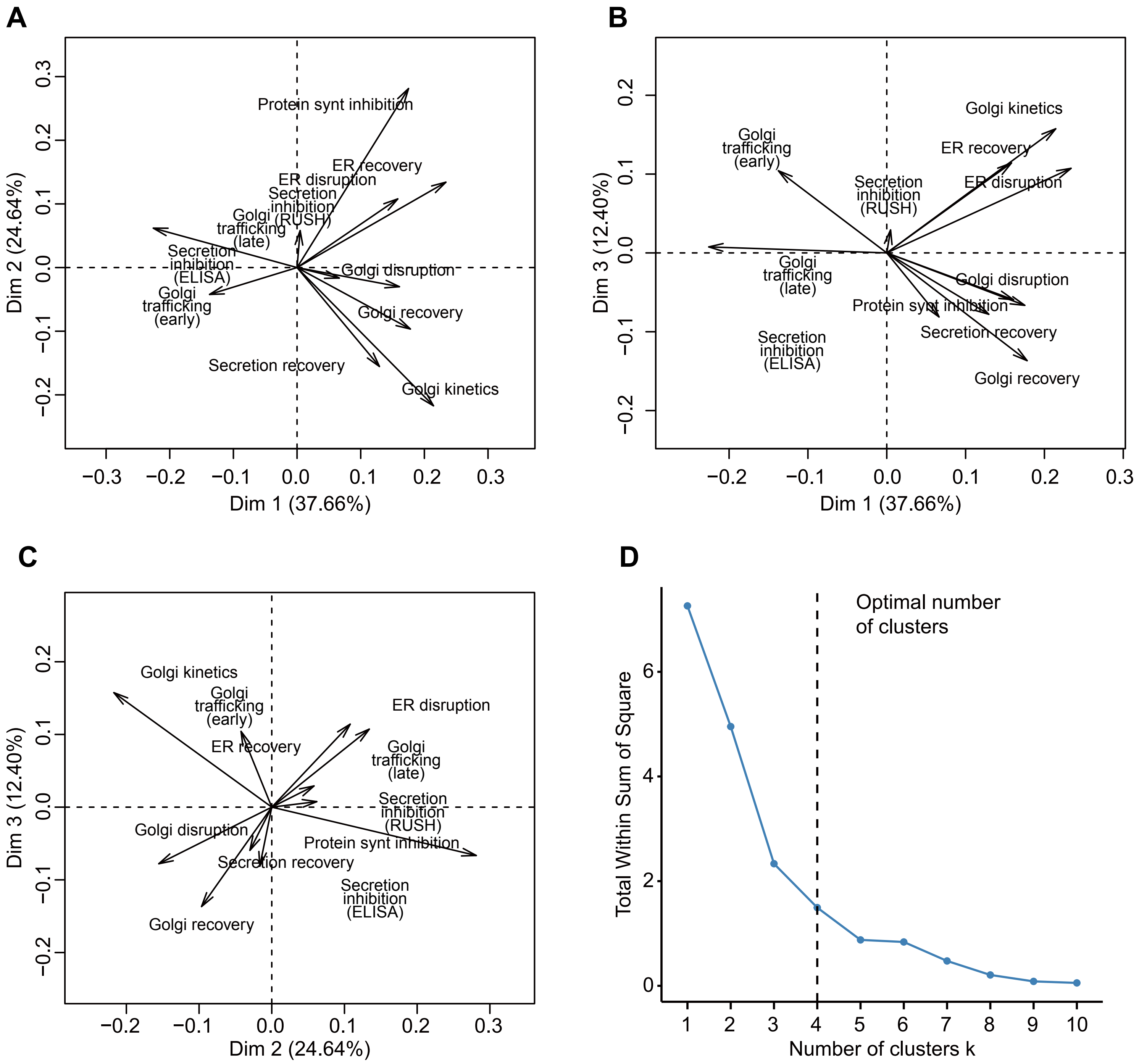

Figure S8. Variable contribution in principal component analysis. (**A,B,C**) Factor maps represent the contribution of each variable to the 3 main dimensions of a principal component analysis. (**D**) The total within sum of square (WSS) was calculated for K-means clustering with incrementing number of groups, and reported in a plot. The optimal number of clusters was chosen as the one preceding the main knee-point of the curve.

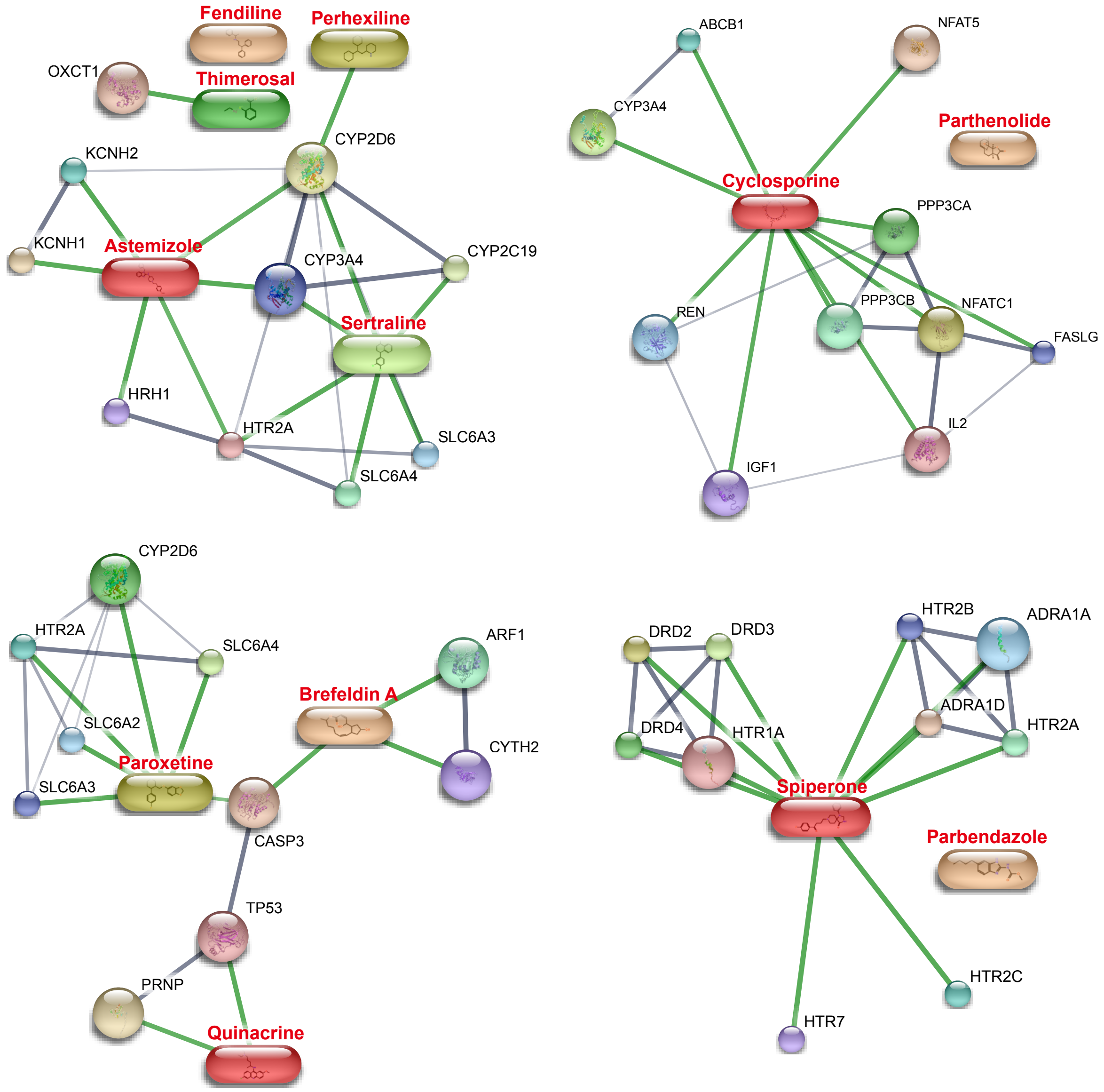

Figure S9. STITCH analysis of potential protein targets shared by identified secretion inhibitors. Inhibitors were analyzed for protein interaction networks using the STITCH database (stitch.embl.de) according to the clusters established by PCA depicted in Figure 8.

Supplemental methods

Temperature controlled secretion assay. U2OS KDEL-strep/SBP-GFP cells were seeded in black 384-well imaging plates and let adapt for 24 h to normal culture condition (37 °C). On the second day, the plates were first transferred to 20 °C for 4 h and then the medium was replaced with complete medium containing 40 μM biotin (20 °C) and maintained for another 24 h at 20 °C before selected secretion inhibitors were added to cells at two different concentrations, BFA (2.5 or 5 µg/mL) was used as a positive control. Cells were incubated with the compounds for 4 h at 20 °C before the plates was transferred to 37 °C. Following incubation of the cells for 4 h, medium was discarded and cells were fixed with 4% PFA containing 2 µg/mL Hoechst 33342 overnight at 4 °C. Next the cells were washed, superseded with 50 μL of PBS and subjected to automated image acquisition and subsequent image analysis.

Detection of cytokines secretion from inducible immortalized dendritic cells (iniDCs). The iniDCs were a kind gift by Dr. Cornelia Richter (Technische Universitaet Dresden, Germany). Culture and de-induction of iniDCs to get de-iniDCs with the characteristics of primary DCs were performed as previously published by Richter et al.(*1*). IniDCs were seeded in 12-well plates (0.5 million/well) and de-induced for 3 days to get functional de-iniDCs. De-iniDCs were pretreated by selected secretion inhibitors for 4 h at two different concentrations, and BFA (2.5 or 5 µg/mL) was used as a positive control. After the treatment cells were stimulated for further 24 h with LPS (2 μg/mL in 1 mL medium) before supernatants were collected for ELISA. ELISA-mediated detection of IL-6 and TNFα was performed with commercial kits (#431302, #430902 from Biolegend) following the manufacturer's protocols.

Supplemental references

1. C. Richter et al., Generation of inducible immortalized dendritic cells with proper immune function in vitro and in vivo. PLoS One 8, e62621 (2013).
